# An Unexpected Outcome of Good Vision After a Penetrating Corneal Trauma by a Bayonet

**DOI:** 10.7759/cureus.22415

**Published:** 2022-02-20

**Authors:** Anastasia Tsiogka, Iordanis Georgiou

**Affiliations:** 1 Ophthalmology, 401 General Military Hospital, Athens, GRC

**Keywords:** surgical treatment, military trauma, bayonet, corneal trauma, ocular trauma

## Abstract

Ocular trauma is a major cause of visual impairment. Corneal injuries can range from minor and insignificant to major and vision-threatening. This report describes the case of a healthy 21-year-old soldier who presented to the emergency department with a major ocular trauma to his left eye caused by a bayonet. The bayonet had penetrated the cornea and reached the anterior chamber. Under general anesthesia, we washed the anterior chamber, filled it with viscoelastic, and sutured the trauma. Visual acuity was 10/10 in his left eye after the surgery. We report this case because there is a limited number of patients with penetrating corneal bayonet injuries reported in the literature and to emphasize the importance of immediate and correct treatment for a good visual outcome.

## Introduction

Ocular trauma is an important, preventable cause of visual impairment worldwide [1,2]. Eye trauma accounts for about 3% of all emergency department visits. The incidence of corneal abrasion is higher among people of working age, affects more males than females, and occurs more frequently in individuals with low socioeconomic status and low educational level [2,3]. Corneal injuries range from minor and insignificant to permanent visual impairment with long-term disabilities. The more severe corneal injuries include ocular surface burns and penetrating trauma [2].

Preoperative evaluation is important for the examination of the etiological agent and the extent of the injury. Understanding the wound architecture is crucial during surgical management to use an appropriate suturing technique for restoring anatomical integrity of the globe and minimizing corneal scarring. Postoperative complications include epithelial ingrowth, wound leakage, and excessive corneal astigmatism [4].

Here, we report the case of a young soldier who suffered a severe corneal trauma with a bayonet. We would like to highlight the importance of immediate and appropriate treatment for a good visual outcome.

## Case presentation

A 21-year-old male was referred to the emergency department with an ocular trauma in his left eye caused by a bayonet. His medical and family histories were unremarkable. Best-corrected distance visual acuity was 10/10 sc in the right eye and 1/10 with pinhole correction in the left eye (Snellen chart). The intraocular pressure of his right eye was 10 mmHg, and we could not measure the intraocular pressure of his left eye. Ocular motility was normal. The eyelids, eyelashes, and conjunctiva appeared normal. There was a full-thickness corneal wound in his left eye expanding between the 3 and 9 o’clock positions in the cornea . The pupil of his right eye was normal, round, and reactive to light, with no afferent pupillary defect. He had a small iridodialisis at the 3 o’clock position in the cornea of his left eye. The intraocular lens of his left eye was in position. Fundus examination of right eye was normal, but fundus examination of the left eye was not possible.

**Figure 1:**
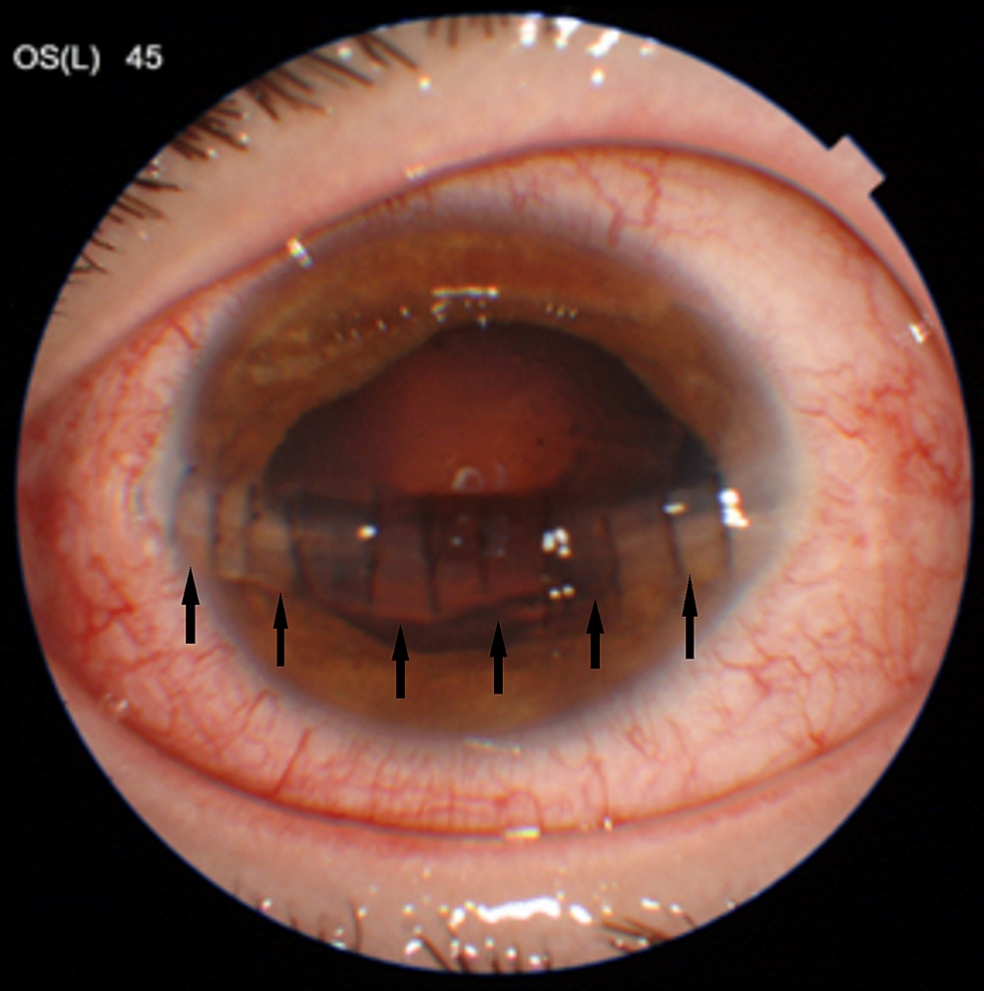
Anterior segment photograph depicting the stitched corneal treatment for the patient (black arrows).

A CT examination excluded possible intraocular foreign bodies. Under general anaesthesia, we washed the anterior chamber, filled it with viscoelastic, and sutured the wound using 12 10.0 Nylon corneal loose stitches, 1 9.0 Nylon sclerocorneal stitch, and 2 8.0 Nylon scleral stitches. With a side port we filled the anterior chamber with balanced salt solution, put intracameral cefuroxime, and administered subconjuctival steroids. On the first postoperative day, his visual acuity was 1/10 with pinhole correction and no sign of infection (Figure 1).

**Figure 2:**
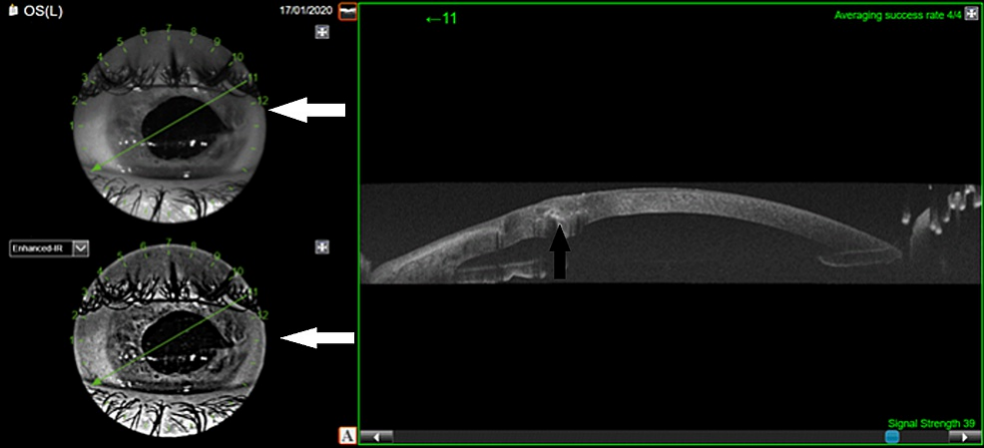
Anterior segment OCT demonstrating a good anatomic (black arrow) and aesthetic (white arrows) outcome. OCT: optical coherence tomography

No other additional surgeries or subsequent ocular complications occurred after the initial treatment for the patient. Six months after the surgery, all of the corneal sutures were removed, and our patient exhibited excellent visual acuity of 10/10 sc. The structure of the wound edges was analyzed with anterior segment optical coherence tomography, demonstrating a good anatomic and aesthetic result (Figure 2).

## Discussion

Corneal laceration and perforation can be accidental, with a majority of injuries resulting from blunt or sharp objects [3]. Ocular trauma occurs in younger people who are often working adults, with many being unable to return to their previous activity with a significant impact on their socioeconomics [2].

The time between injury and surgical intervention is thought to play a crucial role. Visual acuity and slit-lamp examination are important for the recognition and diagnosis of such traumas. Slit-lamp examination, in a full-thickness corneal perforation, can reveal a misshapen iris, hyphema, and a shallow anterior chamber [3]. A CT of the orbit should be obtained when the history or physical examination suggests the possibility of such an injury [3]. Emergent management of such injuries is important to relieve pain and prevent further damage to the eye [5].

We report this case because there is a limited number of patients with penetrating corneal bayonet injuries reported in the literature [4] and to emphasize the importance of immediate and correct treatment for a good visual outcome.

## Conclusions

This is a case of an excellent visual outcome after surgery for a corneal perforation with a bayonet. Patients presenting with traumatic ocular injuries may not show visual acuity improvement. In this case, the patient presented with 1/10 vision with pinhole correction that improved to 10/10 with pinhole correction six months after the surgery. No other additional surgeries or ocular complications occurred after the initial treatment for this patient. Immediate and correct treatment was crucial for the patient’s good visual outcome.
